# Predicting Stunting Beyond Borders: Lessons From an Ethiopian Model and Pathways for Global Application

**DOI:** 10.1002/hsr2.71689

**Published:** 2026-01-11

**Authors:** Bunga A. Paramashanti

**Affiliations:** ^1^ Research Center for Public Health and Nutrition National Research and Innovation Agency (BRIN) Jakarta Indonesia

Dear Editor,

The study by Ahmed et al., “Development and Validation of a Predictive Model for Individual Risk Prediction of Stunting in Ethiopia: A Predictive Modeling Study” [1], provides an important contribution to child nutrition research. Through the development and internal validation of a nomogram‐based model using national survey data, the authors advance individualized risk prediction of stunting among children under two, a critical issue in settings with persistent undernutrition. This letter reflects on key insights from their work, discusses methodological aspects and potential improvements using advanced predictive methods, and considers how similar models might be applied in other countries facing high and persistent rates of stunting.

Ahmed et al. [1] effectively translate Demographic and Health Survey (DHS) data into a practical predictive tool, demonstrating methodological rigor using LASSO regression, multilevel modeling, and bootstrapping‐based internal validation. The study exemplifies a shift from descriptive epidemiology to predictive analytics, identifying children at elevated risk rather than merely documenting prevalence, which marks an important step toward precision public health. Their nomogram, comprising eight readily measurable predictors, including maternal education, child age and sex, feeding practices, and residence, offers a simple and interpretable tool for frontline workers to guide targeted interventions. Incorporating Decision Curve Analysis further bridges statistical modeling and programmatic decision‐making, linking prediction thresholds to policy‐relevant action. These lessons show how national survey data can be leveraged to prioritize children most at risk, strengthening early detection and intervention strategies.

Despite these strengths, several methodological limitations warrant reflection. The cross‐sectional nature of DHS data constrains causal inference and temporal prediction, as stunting reflects cumulative nutritional deficits. Longitudinal data would better capture growth trajectories. Internal validation through bootstrapping may overestimate generalizability, particularly in Ethiopia's diverse geographic and socioeconomic context, highlighting the need for external validation across regions and time points. The predictor set is limited to DHS variables, omitting key determinants such as maternal diet, household food security, and sanitation, which could improve predictive accuracy. Additionally, some predictors (e.g., marital status, residence) may proxy structural inequities rather than causal mechanisms, suggesting that future models should emphasize modifiable factors like dietary diversity and feeding practices to guide actionable policies. Predictive tools must also be used ethically, ensuring referral systems and counseling accompany the identification of high‐risk children to avoid stigmatization.

Future research could leverage advanced predictive techniques to improve accuracy, interpretability, and context relevance. Machine learning (ML) methods such as Random Forests, Gradient Boosting (XGBoost, LightGBM), and Artificial Neural Networks capture nonlinear interactions among environmental, socioeconomic, and maternal‐child factors that linear models may overlook, with AUC values exceeding 0.90 reported in Egypt [2]. Bayesian and hierarchical models allow integration of prior evidence and multilevel structures, estimating individual‐ and community‐level effects. Spatial Bayesian models have revealed subnational‐level stunting heterogeneity in Rwanda [3] and Indonesia [4], which national averages often mask. Ensemble frameworks like Super Learner combine multiple algorithms to balance interpretability and predictive power, and explainable AI tools (e.g., SHAP, LIME) can clarify variable contributions, while integrating geospatial and climate data could strengthen early warning systems in vulnerable regions. These approaches point to a future of hybrid, explainable, and context‐aware predictive models that inform both clinical and policy decision‐making.

The Ethiopian model also offers a blueprint for other low‐ and middle‐income countries with persistent stunting, though contextual adaptation is essential. Predictors vary across regions due to differences in diet, maternal health, sanitation, social protection, and climate exposure. Countries such as Indonesia, Niger, and Madagascar could adapt and validate similar models using locally relevant variables, including dietary diversity, maternal nutrition, and access to health services. Harmonizing modeling frameworks and promoting open‐source algorithms would allow recalibration with national data while ensuring comparability. Importantly, predictive models should complement, not replace, community‐based growth monitoring and multi‐sectoral interventions, forming hybrid surveillance systems where analytics guide resource allocation and community systems ensure equitable implementation.

Overall, Ahmed et al. [1] provide a methodologically sound, interpretable, and policy‐relevant predictive model for stunting risk. Expanding this work globally requires external validation, inclusion of modifiable predictors, and integration of advanced machine learning and Bayesian techniques. Predictive analytics can optimize resource allocation in stunting‐prone regions, but their impact ultimately depends on embedding these models within holistic, equity‐driven nutrition systems that address social, environmental, and structural determinants of undernutrition.

## Author Contributions

**Bunga A. Paramashanti:** conceptualization, writing – original draft, writing – review and editing.

## Ethics Statement

This letter to the editor did not involve the collection of new data or human participants and therefore did not require ethical approval.

## Conflicts of Interest

The author declares no conflicts of interest.

## Transparency Statement

The author affirms that this letter is an honest, accurate, and transparent account of the topic discussed; no important aspects have been omitted, and any discrepancies from the referenced study are clearly noted.

## Data Availability

Data sharing does not apply to this article as no data sets were generated or analyzed during the current study.
